# Characteristics of Extremely Preterm Infants Undergoing Procedural Closure of Patent Ductus Arteriosus: A Retrospective Cohort Study

**DOI:** 10.1155/ijpe/5558870

**Published:** 2025-11-28

**Authors:** N. Jayakumar, G. Uthayakumaran, S. M. Boyd, J. Mervis, R. Halliday, A. Webb, H. Popat

**Affiliations:** ^1^Grace Centre for Newborn Intensive Care, The Children's Hospital at Westmead, Westmead, Australia; ^2^The University of Sydney, Sydney, Australia; ^3^Cerebral Palsy Alliance, Research Institute, Sydney, Australia; ^4^NHMRC Clinical Trial Centre, Camperdown, Australia

**Keywords:** infants, neonates, patent ductus arteriosus, preterm, surgical ligation, transcatheter device closure

## Abstract

**Background:**

Patent ductus arteriosus (PDA) is the most common cardiac lesion in preterm newborns. When medical management of a hemodynamically significant PDA is unsuccessful or contraindicated, infants are referred for either transcatheter device closure (TCDC) or surgical ligation. Our objective was to describe the characteristics and outcomes of these infants.

**Methods:**

A retrospective cohort study of infants ≤ 30 weeks' gestation undergoing either TCDC or surgical ligation for PDA from January 2009 to April 2023 was undertaken, in a surgical neonatal intensive care unit. Baseline demographics, echocardiographic data, procedural complications, and neonatal outcomes were obtained.

**Results:**

A total of 136 infants were included. At the time of referral for PDA closure, infants were 5–145 days old, with a corrected gestational age of 24–50 weeks and PDA diameter of 1.5–4.1 mm. TCDC of PDA was performed in 15 neonates compared with 121 neonates who underwent surgical ligation. Procedural complications and important neonatal outcomes were similar for both groups. While the number of infants undergoing TCDC is increasing, there is a decreasing trend in the total number of surgical PDA closures.

**Conclusions:**

This study demonstrates that there is variability in the preclosure demographics and echocardiography characteristics of infants ≤ 30 weeks' gestation referred for procedural PDA closure.

## 1. Introduction

Patent ductus arteriosus (PDA) remains the most common cardiac lesion in premature newborns and its incidence is inversely related to gestational age [1, 2]. Persistent PDA, defined as failure of the PDA to close within the first 72 hours of life, is reported in 10% of newborns born at 30–37 weeks' gestation compared with 90% of neonates born between 25–28 weeks' gestation [3, 4].

With increasing survival of extremely preterm newborns, the overall incidence of PDA is increasing. While there is no single parameter that defines a hemodynamically significant PDA (hsPDA), a combination of clinical assessment for signs and symptoms of pulmonary overcirculation and systemic hypoperfusion as well as echocardiographic indices such as ductal size and Doppler pattern forms the current gold standard for evaluation of a hsPDA [5–7]. The presence of hsPDA may be associated with prolonged ventilation, impaired renal function, intraventricular hemorrhage, periventricular leukomalacia, pulmonary hemorrhage, bronchopulmonary dysplasia, necrotizing enterocolitis, and retinopathy of prematurity [7, 8]. The associations of hsPDA in preterm infants in the literature are well described, including both mortality and significant morbidity [8]. Despite this, there is little consensus as to which patients require treatment and regarding when treatment should optimally occur.

First-line management for a hemodynamically significant PDA is most commonly pharmacological using either indomethacin, ibuprofen, or paracetamol [9, 10]. If pharmacological management is unsuccessful, referral for closure via surgical ligation or a transcatheter device closure is considered [2, 8]. The aim of this study is to describe the characteristics and outcomes of preterm infants referred for either TCDC or surgical ligation of PDA in a single institution over a 15-year period. This information may assist in identifying management trends as well as factors associated with poor outcomes in this population.

## 2. Methodology

### 2.1. Study Population

The study included all preterm infants ≤ 30 weeks' gestation who underwent surgical PDA ligation or TCDC of PDA from January 2009 to April 2023 at The Grace Centre for Newborn Intensive Care, Westmead Australia. This is one of two referral centers for procedural PDA management in New South Wales, Australia's most populous state with a population of 8 million people. Infants were included irrespective of whether or not they had received prior pharmacological treatment for ductal closure which was at the discretion of the treating neonatologist.

### 2.2. Exclusion Criteria

Those with birth locations outside Australia were excluded as were those with associated major extracardiac anomalies, congenital cardiac defects, or genetic diagnoses. These infants were excluded on the basis that this study is primarily focused on analyzing trends and outcomes of PDA management in the preterm population.

### 2.3. Data Acquisition

Data were obtained from The Neonatal Intensive Care Units' Data Registry (NICUS), which is an ongoing prospective statewide audit of infants admitted to 10 NICUs (eight perinatal centers and two children's hospitals) in New South Wales. Definitions used and data quality have been described elsewhere [11]. Baseline demographic data, clinical characteristics of the infants prior to duct closure, and neonatal outcomes were obtained for each infant from the NICUS database. Preclosure transthoracic echocardiograms were reviewed by a single observer (G.U.). Ductal diameter, direction of ductal shunt, and ductal Doppler pattern were recorded. The PDA diameter was measured on 2D imaging at the narrowest portion before its entry into the main pulmonary artery, during the period of maximal flow [12]. The size of the ductus was expressed as an absolute value as measured in millimeters (mm) and indexed to the body weight of the infant, calculated in millimeters/kilogram (mm/kg). The weight used for the indexed calculation was the pre-procedural weight [13]. The direction of shunt across the PDA was defined by the color Doppler profile, reported as either left to right, bidirectional, or right to left. The Doppler profile was defined as either continuous or pulsatile [12]. The presence, size, and direction of an atrial shunt were also recorded as small, moderate, and large defects being defined as 5, 6–8, and > 9 mm, respectively. Data will be made available from the authors upon reasonable request.

## 3. Statistical Methods

Infant demographics and characteristics, echocardiogram data, and neonatal outcomes were summarized with medians and ranges for continuous variables and frequencies for categorical variables. Differences between the TCDC and ligation groups were investigated using chi-squared tests of association for categorical variables, ANOVA or nonparametric Kruskal–Wallis tests for continuous variables, and proportional hazards regressions for length of stay variables. Poisson regression was conducted to test for any significant trend in the number of procedures over time. Statistical significance was set at *p* < 0.05, and no adjustments for multiple comparisons were applied, as analyses were considered exploratory. All analyses were conducted in R v4.3.1 [14].

## 4. Results

A total of 136 infants were referred for TCDC or surgical ligation of PDA over the 15-year study period. The median gestation at birth for all infants referred was 25 weeks' gestation (ranged from 23 to 29 weeks' gestation), and the median birth weight of all infants referred was 814 g (ranged from 450 to 1557 g). A total of 15 neonates underwent TCDC of PDA compared with 121 neonates who underwent surgical ligation of PDA. The baseline demographics of these infants are described in Table 1. The infants in the TCDC group were more mature at birth (27 vs. 25 weeks), comprised a significantly higher proportion of males (80% vs. 47%), and were significantly more likely to be SGA (60% vs. 23%). All infants received at least one dose of corticosteroids antenatally.

The characteristics of the infants prior to surgical closure are described in Table 2. There is a broad range in all infant characteristics at the time of referral, including the gestational age at closure and pre-procedural weight, which ranged from 24 to 50 weeks' corrected and 529–5640 g, respectively. The median gestation at the time of surgical closure in the TCDC group was 33 weeks' with a median weight of 1430 g compared with 29 weeks' and 1005 g in the surgical ligation group. At the time of closure, the infants within the TCDC group were significantly older (by both chronological age and GA), of higher weight, and significantly less likely to be managed with invasive mechanical ventilation prior to the procedure.

A subgroup analysis (Table 3) comparing infants between January 2017 and April 2023 was performed to further investigate the two groups following the introduction and availability of the TCDC device. The gestational age, weight, and feeding status prior to procedural closure of the PDA are marginally statistically significant.

The duct and shunt echocardiographic features obtained at admission to the surgical unit are described in Table 4. The median absolute PDA diameter was 2.5 and 2.6 mm in the TCDC and ligation group, respectively. All infants had an absolute PDA which measured ≥ 1.5 mm. The infants within the TCDC group had significantly lower PDA diameter when indexed to body weight and marginally significantly greater Doppler peak systolic velocity.

**Figure 1:**
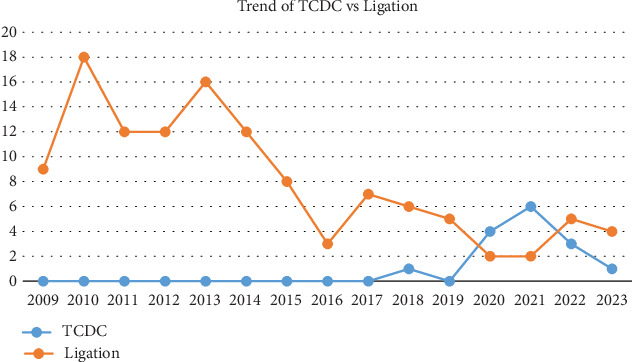
Trends of TCDC versus surgical ligation from January 2009 to April 2023.

Figure 1 demonstrates the number of TCDC and surgical ligations performed each year between 2009 and 2023.  Table 5 demonstrates the Poisson regression results. The current number of PDA ligations from the 2017–2023 epoch is approximately half that of the 2009–2016 epoch, while the number of infants undergoing TCDC is increasing overall. The average number of total procedural PDA closures per year after 2017 is only 60% of the average number before. Therefore, there is a decrease of 40% in the average number of procedural PDA closures per year after 2017.

The short- and long-term outcomes for the infants referred are outlined in Table 6. Four infants within the surgical ligation group were initially planned to undergo TCDC; however, TCDC was abandoned in the catheter laboratory in favor of surgical ligation. In these four cases, device closure, though planned, was not attempted due to the PDA being assessed as too large for the device available at that time. These infants are accounted for in the ligation group. Two infants developed complications following TCDC. One infant had post PDA closure syndrome, and one infant developed ventilator-associated pneumonia requiring prolonged intubation. There were 31 procedural complications in 28 patients identified in the surgical ligation group. The most common complication was vocal cord palsy (15/28). Two infants required reintervention with repeat surgical ligation. The first infant developed left bronchial compression related to the PDA ligation clip, and the other infant had a hemodynamically significant residual PDA requiring repeat ligation. Morbidity between the two groups was similar. Furthermore, there were no significant differences in secondary neonatal outcomes of BPD, home oxygen requirement at discharge, IVH ≥ Grade II, and NEC between the groups.

## 5. Discussion

This is a single-center retrospective cohort study describing the baseline infant demographics, preclosure clinical characteristics, and key secondary outcomes for infants referred for either TCDC or surgical ligation of PDA following birth at ≤ 30 weeks' gestation. This study demonstrates a variability in the baseline, clinical, and echocardiographic characteristics of infants undergoing procedural closure of PDA. This reflects the heterogeneity of this group of patients and the lack of systematic use of clinical and echocardiographic scoring systems to aid clinical decision-making. This study demonstrated that the TCDC group was older and more mature, and TCDC is a relatively contemporary procedure, with it being performed in this center from 2017. Furthermore, we postulate that neonatologists are possibly less aggressive in treating PDA in the extreme preterm population, with a 40% reduction in procedural PDA closures from 2017 demonstrated by this study.

The study further contributes to the growing literature supporting a trend of less surgical ligation overall and increasing device closure of PDA in preterm infants, which has been observed internationally [8, 15]. O'Byrne et al. (2017) evaluated the trends in transcatheter closure compared with surgical ligation of PDA in 44 neonatal intensive care units across the United States, which concluded that from 2007 to 2017 there was a decreasing number of surgical PDA closures in comparison to rising rates of transcatheter device closure. However, this study did not review short-term outcomes [16]. A more recent study by Shah et al. concluded there had been an overall decrease in surgical ligation and an increase in device closure of PDA among preterm infants 22–30 weeks' gestation between 2014 and 2021 [17]. While this study retrospectively included 2014 infants and provided detailed demographics and clinical characteristics including PDA medical management, the study was limited by an absence of echocardiographic data. Findings from Shah et al. were consistent with those observed in our study, in that infants undergoing surgical ligation were less mature and weighed less at the time of the procedure when compared to those undergoing device closure [17]. Similarly, from a global perspective of PDA management, a recent publication in 2023 by Sathanandam et al. reports a similar trend [15]. This comprehensive review also demonstrates that in preterm infants born at ≤ 26 weeks and birth weight < 1000 g who experience hsPDA requiring mechanical ventilation, there is an increasing trend toward TCDC compared with surgical ligation, with an overall reduction in interventional PDA closure from 2007 to 2021 [15]. Overall, the current body of literature supports the main findings of this single-center study. This trend is likely contributed to by advancements in catheter technology including the availability of a device designed specifically for preterm infants, as well as increasing evidence of safety and efficacy of the procedure in small infants [18, 19]. Additionally, there is likely increasing confidence among neonatologists to refer infants for the TCDC procedure and the subgroup analysis performed in the study compares features that may be considered to determine the procedure of choice for the infant.

The rate of adverse neonatal outcomes, including NEC, CLD, and IVH (≥ Grade 2), in this cohort remains higher than in infants matched for gestational age who did not undergo surgical or transcatheter device closure of PDA. The Australian and New Zealand Network (ANZNN) reported rates of NEC in babies < 28 weeks registered at Level III units between 2012 and 2021 of 5.8%–9.1%, compared with 14% in the infants < 30 weeks who underwent procedural PDA closure in this study. Rates of CLD in Level III units as reported by ANZNN in infants < 31 weeks in 2021 were 35.4%, compared with 55% in all infants referred in our study, 53% in the TCDC group and 58% in the ligation group [20]. We postulate that the presence of a longstanding hsPDA that requires nonpharmacological closure is more likely to be seen in a subset of vulnerable preterm infants who are more likely to have adverse neonatal outcomes.

This study reported the admission echocardiogram data of the infants referred for nonpharmacological closure used to assess hemodynamic significance. This included the absolute PDA diameter, PDA diameter indexed to body weight, direction of PDA shunt, and PDA Doppler profile, as well as the presence, size, and direction of atrial shunt. A number of PDA scoring tools have been published in an attempt to aid rational and systematic patient selection for PDA intervention [21, 22]. More recently, a PDA score encompassing detailed echocardiographic markers for adjudication of hemodynamic significance in infants < 27 weeks has been described by Giesinger et al. [23]. The Iowa PDA score for evaluation of hsPDA incorporates pulmonary vein D wave velocity (cm/s), mitral valve E wave velocity (cm/s), isovolumetric relaxation time (ms), left atrial to aortic (LA/Ao) ratio, left to right ventricular output ratio, and aorta or peripheral arterial Doppler flow profile. Although a reduction in the composite outcome of death prior to 36 weeks or severe BPD was observed in the epoch utilizing this assessment tool [23], detailed scoring systems of this type have not been evaluated in a randomized trial setting. Future clinical trials should consider prospective evaluation of these scoring systems in an attempt to better target PDA therapy, including nonpharmacological closure, given the well-described limitations of the existing PDA literature [24].

A limitation of this study is that the prolonged study period encompasses a period of significant change in the approach to screening and the management of preterm hsPDA. Many neonatal centers have shifted from earlier diagnosis and an aggressive treatment approach to a more conservative approach to the management of PDAs with later diagnosis and reduced medical or surgical closure in more recent years [25]. In recent times, surgical closure of the PDA has become less frequent, and transcatheter closure is more common in many centers. This was highlighted in an American clinical report which found fewer evaluations of PDA and a subsequent reduction in medical and surgical closure of hsPDA [26].

Further limitations of this study include the retrospective nature of this study and its inherent limitations which can be addressed with prospective research designs. Additionally, the small sample size of the study remained a limitation, particularly the unequal distribution of infants to the TCDC arm and ligation arm. The infants in the TCDC arm are significantly older, 44 days versus 29 days, which makes comparison of the two groups rather difficult. The baseline demographics and characteristics of the babies undergoing procedural closure that have been described may reflect the factors that are considered when referring for procedural closure and the features that may help to decide on either TCDC or surgical ligation. Inclusion in this study was at the discretion of the treating neonatologist who referred the infant for procedural closure to the surgical center. Therefore, we may not have captured all preterm infants ≤ 30 weeks' gestation with hsPDA within the state. Moreover, a further limitation of the study was the comprehensiveness and completeness of the echocardiographic data available which varied over time due to evolving echocardiographic measures that were obtained to determine ductal significance. Additionally, the retrospective nature of the study precluded the application of a standardized hsPDA scoring tool, representing a missed opportunity to objectively stratify PDA. One example is the SIMPLE scoring system (scoring preterm infants for PDA clinically without echocardiographic evaluation), which uses clinical and risk factors to predict early hsPDA in extremely low birth weight infants [27]. Longitudinal collection of data from this growing population will continue to contribute to further knowledge in this area. Furthermore, our study did not include neurodevelopmental outcomes which are important in understanding the long-term effect of surgical intervention on the PDA.

## 6. Conclusion

TCDC and surgical ligation are undertaken on a population of preterm infants among which there is significant heterogeneity, with a lack of unifying clinical parameters at referral. This study contributes to the growing body of literature highlighting the trend of an increasing proportion of infants undergoing interventional closure via TCDC and a reduction in the number of surgical ligations. There is an opportunity to further refine patient selection for transcatheter and surgical ductal closure in the modern era of management, as well as exploring the role of detailed echocardiographic scoring systems in optimizing patient selection for therapy.

## Figures and Tables

**Table 1:** Demographics of infants referred for procedural PDA treatment.

	**All (** **n** = 136**)**	**TCDC (** **n** = 15**)**	**Ligation (** **n** = 121**)**	**p** **value**
Gestation at birth (weeks), median (range)	25 (23–29)	27 (23–29)	25 (23–29)	0.029
Birth weight, median (range)	814 (450–1557)	890 (479–1220)	813 (450–1557)	0.438
Male sex, *N* (%)	69 (51%)	12 (80%)	57 (47%)	0.026
Small for gestational age, *N* (%)	37 (27%)	9 (60%)	28 (23%)	0.005
Antenatal steroids, any, *N* (%)	136 (100%)	15 (100%)	121 (100%)	0.999

**Table 2:** Characteristics of infants prior to procedural PDA closure.

	**All (** **n** = 136**)**	**TCDC (** **n** = 15**)**	**Ligation (** **n** = 121**)**	**p** **value**
Chronological age at time of closure (days), median (range)	30 (5–145)	44 (14–96)	29 (5–145)	0.007
Gestational age at time of closure (weeks), median (range)	30 (24–50)	33 (29–42)	29 (24–50)	0.001
Weight at time of closure (grams), median (range)	1055 (529–5640)	1430 (800–3690)	1005 (529–5640)	< 0.001
Medical treatment (yes), *N* (%)	124 (91%)	14 (93%)	110 (91%)	0.999
Invasive mechanical ventilation (yes), *N* (%)	83 (61%)	5 (33%)	78 (64%)	0.026
Inotropes (yes), *N* (%)	3 (< 1%)	0	3 (< 1%)	0.999
Feeding status prior to referral (any enteral feeds), *N* (%)	83 (61%)	13 (87%)	82 (68%)	0.231

**Table 3:** Subgroup analysis of the demographics and characteristics of infants undergoing procedural PDA closure between January 2017 and April 2023.

	**T** **o** **t** **a** **l** = 46	**TCDC (** **n** = 15**)**	**Ligation (** **n** = 31**)**	**p** **value**
Gestation at birth (weeks), median (range)	26 (23–29)	27 (23–29)	25 (24–29)	0.092
Birth weight, median (range)	851.5 (450–1557)	890 (479–1220)	843 (450–1557)	0.527
Male sex, *N* (%)	27 (66%)	12 (80%)	15 (48%)	0.085
Small for gestational age, *N* (%)	17 (37%)	9 (60%)	8 (26%)	0.054
Antenatal steroids, any, *N* (%)	46 (100%)	15 (100%)	31 (100%)	0.999
Chronological age at time of closure (days), median (range)	34 (8–96)	44 (14–96)	31 (8–96)	0.052
Gestational age at time of closure (weeks), median (range)	30 (27–42)	33 (29–42)	30 (27–41)	0.021
Weight at time of closure (grams), median (range)	1278 (574–3690)	1430 (800–3690)	1158 (574–3672)	0.018
Medical treatment (yes), *N* (%)	44 (96%)	14 (93%)	30 (97%)	0.999
Invasive mechanical ventilation (yes), *N* (%)	23 (50%)	5 (33%)	18 (58%)	0.208
Inotropes (yes), *N* (%)	1 (2%)	0	1 (3%)	0.999
Feeding status prior to referral (any enteral feeds), *N* (%)	11 (24%)	13 (87%)	9 (29%)	< 0.001

**Table 4:** Admission echocardiogram data at surgical center of infants prior to nonpharmacological PDA closure.

	**All (** **n** = 136**)**	**TCDC (** **n** = 15**)**	**Ligation (** **n** = 121**)**	**p** **value**
Absolute PDA diameter (mm), median (range)	2.5 (1.5–4.1)	2.5 (2–3.5)	2.6 (1.5–4.1)	0.609
PDA diameter indexed to body weight (mm/kg), median (range)	2.42 (0.70–6.23)	1.61 (0.83–3.38)	2.60 (0.70–6.23)	< 0.001
Direction of shunt: Left to right, *N* (%)	130 (100%) *n* = 130	15 (100%) *n* = 15	115 (100%) *n* = 115	0.999
PDA Doppler profile–pulsatile yes, *N* (%)	113 (92%) *n* = 123	15 (100%) *n* = 15	98 (91%) *n* = 108	0.609
Doppler peak systolic velocity (m/s), *N* (%)	2.3 (1.0–3.9) *n* = 118	2.6 (1.2–3.6) *n* = 15	2.25 (1.0–3.9) *n* = 103	0.052
Presence of atrial shunt (yes), *N* (%)	121 (92%) *n* = 131	13 (87%) *n* = 15	108 (93%) *n* = 116	0.321
Size of atrial shunt, *N* (%)				
Small	93 (78%)	11 (79%)	82 (77%)	0.730
Moderate	27 (23%) *n* = 120	2 (14%) *n* = 13	25 (23%) *n* = 107	
Direction of atrial shunt left to right, *N* (%)	118 (98%) *n* = 120	13 (100%) *n* = 13	105 (98%) *n* = 107	0.999

**Table 5:** Poisson regression results.

	**Total procedures**	**p** ** value**	**Ligation**	**p** ** value**	**TCDC**	**p** ** value**
Overall time trend in number of procedures per year	—	< 0.001	—	< 0.001	—	< 0.001
Mean ratio of post 2017 to 2017 or earlier number of procedures per year	0.60 (0.41, 0.87)	0.007	0.37 (0.23, 0.57)	< 0.001	NA	

**Table 6:** Neonatal outcomes.

	**All (** **n** = 136**)**	**TCDC (** **n** = 15**)**	**Ligation (** **n** = 121**)**	**p** **value**
Procedural complications, *N* (%)	30 (22%)	2 (13%)	28 (23%)	0.520
Death prior to discharge (yes), *N* (%)	12 (9%)	2 (13%)	10 (8%)	0.622
Respiratory support (days), median (range)	77.29 (14.80–252.95)	70.29 (56.33–168.22)	77.33 (14.8–252.95)	0.719
Postnatal steroids (yes), *N* (%)	52 (38%)	4 (27%)	48 (40%)	0.407
BPD (yes), *N* (%)	75 (55%)	8 (53%)	70 (58%)	0.787
Home oxygen (yes), *N* (%)	41 (30%)	4 (27%)	37 (31%)	0.999
IVH >/= Grade 2 (yes), *N* (%)	35 (26%)	4 (27%)	31 (26%)	0.999
NEC (yes), *N* (%)	19 (14%)	2 (13%)	17 (14%)	0.999
Length of stay at GNIC (days), median (range)	4.76 (0.42–253.7)	3.00 (0.42–35.93)	4.89 (1.07–253.7)	0.015
Hospital length of stay (days), median (range)	120.21 (14.83–271.90)	101.71 (56.30–189.70)	120.42 (14.83–271.90)	0.344

Abbreviations: GNIC, Grace Newborn Intensive Care; IVH, intraventricular hemorrhage; NEC, necrotizing enterocolitis.

## Data Availability

The data that support the findings of this study are available on request from the corresponding author. The data are not publicly available due to privacy or ethical restrictions.
